# Laser Displacement Sensors for Wind Tunnel Model Position Measurements

**DOI:** 10.3390/s18124085

**Published:** 2018-11-22

**Authors:** Matthew Kuester, Nanyaporn Intaratep, Aurélien Borgoltz

**Affiliations:** Kevin T. Crofton Department of Aerospace & Ocean Engineering, Virginia Tech, Blacksburg, VA 24060, USA; nintarat@vt.edu (N.I.); aborgolt@vt.edu (A.B.)

**Keywords:** laser displacement sensors, wind tunnel testing, CMOS line camera

## Abstract

Wind tunnel measurements of two-dimensional wing sections, or airfoils, are the building block of aerodynamic predictions for many aerodynamic applications. In these experiments, the forces and pitching moment on the airfoil are measured as a function of the orientation of the airfoil relative to the incoming airflow. Small changes in this angle (called the angle of attack, or α) can create significant changes in the forces and moments, so accurately measuring the angle of attack is critical in these experiments. This work describes the implementation of laser displacement sensors in a wind tunnel; the sensors measured the distance between the wind tunnel walls and the airfoil, which was then used to calculate the model position. The uncertainty in the measured laser distances, based on the sensor resolution and temperature drift, is comparable to the uncertainty in traditional linear encoder measurements. Distances from multiple sensors showed small, but statistically significant, amounts of model deflection and rotation that would otherwise not have been detected, allowing for an improved angle of attack measurement.

## 1. Introduction

Wind tunnel testing plays an important role in the development of many aerospace applications. One of the most basic, fundamental types of wind tunnel tests is to measure the forces and moments on a two-dimensional wing section (airfoil). Wind tunnel measurements are often used to validate numerical models, and the two-dimensional force and moment coefficients can be extrapolated to estimate aerodynamic forces and moments on propellers/wind turbines [1] and airplane wings [2].

The aerodynamic forces and moments are inherently sensitive to model geometry and model orientation relative to the incoming airflow. For example, thin airfoil theory [3] predicts that a $1^{\circ }$ change in the angle of attack (the relative angle of the incoming flow relative to the airfoil) will change the lift coefficient (the normalized aerodynamic force in the direction perpendicular to the flow) by 0.11. This means that accurately measuring the angle of attack (α) is critical for airfoil wind tunnel measurements.

The typical setup for these experiments features an airfoil model mounted to a turntable, with the angle of attack measured by a magnetic encoder attached to the turntable. The loading on the airfoil depends on the flow conditions, the size of the model, and the model geometry. For the model and wind tunnel used in this work, lift forces up to 6000 N and pitching moments up to 500 Nm can cause the model to deflect or rotate, creating an angle of attack offset that may not be detected by an encoder. Additionally, model deflections can also cause spanwise non-uniformity in the model surface through twisting and bending, which may alter the aerodynamics.

These challenges have traditionally not been addressed in airfoil wind tunnel tests, but they have been addressed in wind tunnel tests of scaled airplane models. The most common technique is to include accelerometers [4,5] inside of the model; the output of the accelerometers can be integrated to independently track the position and orientation of the model. Multiple accelerometers can be used to track positions/orientations at multiple points on the model, but the accelerometer output needs to be tracked continuously for the duration of the experiment. Photogrammetry techniques [5,6,7,8,9,10] have also been used to visually track marker positions on the model, which can be post-processed to calculate model deflections and deformations. This technique is very useful in aeroelasticity tests, but requires optical access for multiple cameras and significant post-processing to determine the model orientation. Laser interferometry [5] can also be used to track model positions, but the equipment cost and setup time make it impractical for most wind tunnel tests.

The motivation for this work was to identify a secondary way to measure airfoil model positions for wind tunnel applications. After reviewing multiple options, triangulation laser displacement sensors were selected to measure the distance between the airfoil model and the wind tunnel walls. These sensors were selected for a number of reasons: they are commercially available, are self-contained, and do not interfere with other wind tunnel measurements. Laser displacement sensors also provide advantages over accelerometer and photogrammetry techniques because they can detect very small deflections while providing an absolute reference for determining model angle of attack, while these other techniques only provide a relative change in position. The results presented here describe the implementation and integration of these sensors into a low-speed wind tunnel, and how multiple sensors are used to detect changes in position due to aerodynamic loading.

## 2. Experimental Setup

The following subsections describe the experimental setup, the laser displacement sensors installed in the wind tunnel, and previous work in using these sensors to measure wind tunnel model position.

### 2.1. Wind Tunnel Facility and DU96-W-180 Airfoil Model

**Figure 1:**
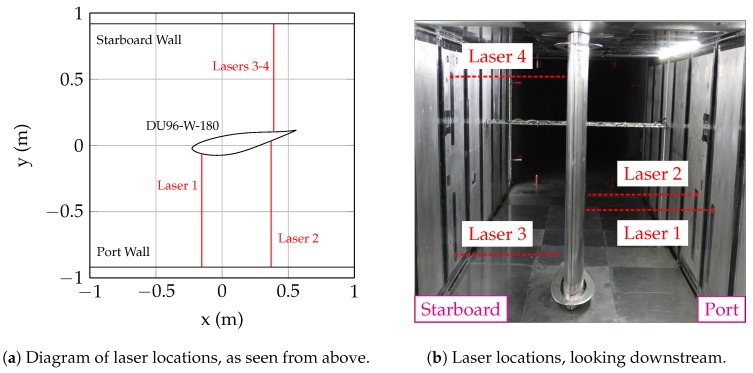
Setup of laser distance sensors and 0.8 m DU96-W-180 in the Virginia Tech Stability Wind Tunnel.

All of the measurements described in this paper were performed in the Virginia Tech Stability Wind Tunnel (VT SWT) [11]. The VT SWT is a closed return, low-disturbance facility capable of reaching freestream air speeds of 75 m/s. The facility is used extensively for wind turbine airfoil measurements; the size and speed capabilities of the tunnel allow for full-scale Reynolds number testing of wind turbine blade sections. The aerodynamic test section (used in this work and shown in Figure 1) is a 7.3 m long rectangular prism, with a 1.85 m × 1.85 m square cross section. The tunnel walls are solid aluminum panels, and the floor is made of aluminum floor/ceiling panels, all supported by a steel outer structure. The tunnel coordinate system is centered in the middle of the test section; the +x direction points downstream (in the direction of the flow), the +y direction points toward the starboard wall, and the +z direction points toward the ceiling.

For this work, a 0.8 m chord DU96-W-180 airfoil model was installed into the test section shown in Figure 1b. This model, which has been used in a number studies at the VT SWT [12,13], was constructed at Virginia Tech by the Aerospace and Ocean Engineering Machine Shop. The model is constructed of 50-mm thick CNC-machined aluminum laminates. The laminates are stacked in the span direction, pinned together, and held in compression by four 9.5 mm threaded rods that run the entire span of the model. The model is rotated to the appropriate angle of attack using an automated turntable in the ceiling, with a maximum holding torque of 1000 Nm. The rotational position of the turntable is measured using a Renishaw LM10 linear magnetic encoder with resolution smaller than 0.004 degrees. The model is attached to a bearing installed underneath the floor that allows the model to rotate freely, but prevents model translation at the floor.

**Figure 2:**
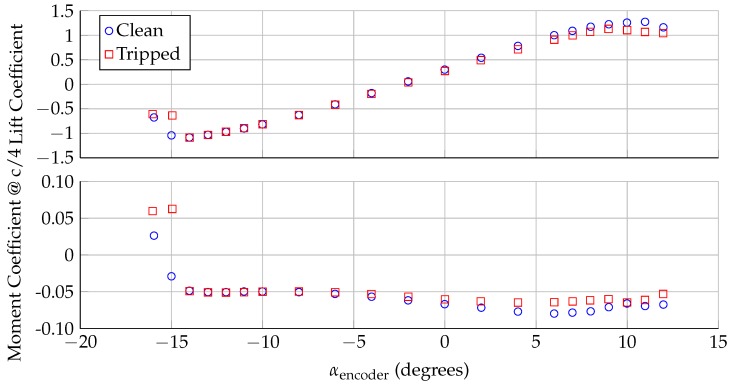
Lift and moment coefficients for the DU96-W-180 at Rec=3.0 million.

During a typical experimental measurement, the model is rotated through a series of angles of attack with the airflow set at a near-constant speed. At each measurement point, the turntable is locked to a set angle of attack. After the flow settles for 3–4 s, pressure transducers measure the pressure on approximately 90 pressure taps, distributed along the chordline of the model near midspan. After acquiring pressure data for 20–40 s, the model is rotated to the next angle of attack. This surface pressure can be integrated to calculate the lift coefficient ($c_{l}$) and the moment coefficient about the quarter chord point ($c_{m}$) [14]. Figure 2 shows the lift and moment coefficients as a function of angle of attack for the DU96-W-180 as measured in the VT SWT at a chord Reynolds number of 3.0 million (freestream air speed of ∼63 m/s). Two different configurations are shown here: clean and tripped. In the tripped configuration, a 0.4 mm tall zig-zag tape is placed near the leading edge of the model, which affects the growth of the boundary layer and changes the aerodynamic loads. By convention, a negative moment coefficients indicates that, if not constrained, the model would tend to rotate to a more negative angle of attack. Both the lift and moment coefficients change with angle of attack, which means that the aerodynamic loading on the model also changes with angle of attack.

### 2.2. Laser Distance Sensors

Four laser distance sensors were installed in the walls of the wind tunnel to track model position/orientation. Acuity AR700-32 laser units were chosen for this application because the span and range of the systems allowed for measurements across a wide range of angles of attack. Each unit consists of a Class 3R laser (670 nm, 5 mW) and a CMOS line camera (43.2 mm long sensors area, 425 mm away from the laser origin). The laser is pointed at a target (in this case, the wind tunnel model). Images from the line camera are processed in real time with an on-board processor to calculate the distance between the laser origin and the target. The sensors can be configured for analog or serial output; for this application, each unit is connected to a data acquisition computer using a RS-232 serial interface. The wind tunnel data acquisition system queries the lasers each time the wind tunnel model is rotated. The standoff distance of these units is 1067 mm, with a measurement span of 812.8 mm. The resolution of the distance measurement is 41 μm, and the quoted linearity of the measurement is ±406 μm.

Figure 1 shows the Acuity AR700-32 units installed in the wall of the wind tunnel. Each sensor is held in place by an extruded aluminum mounting structure on the outside of the wind tunnel. The sensors are mounted flush with the wind tunnel wall, but are not rigidly attached to the wall, which ensures that the laser position is constant and is not affected by wall vibrations and/or deflections. The laser locations were chosen as to not interfere with other instrumentation installed in the wind tunnel walls, while also providing multiple measurements along the span (z) and chord (x) directions. The lasers are numbered for easy identification. Laser 1 is installed in the port wall, approximately 175 mm upstream of the model rotation point. Laser 2 is also installed in the port wall, but approximately 545 mm downstream of Laser 1. Lasers 1 and 2 are at the same span location in the tunnel, approximately 660 mm above the floor (z = −265 mm). Lasers 3 and 4 are installed in the starboard wind tunnel wall, at the same streamwise location as Laser 2. Laser 3 is close to the floor (approximately 200 mm from the floor, z = −725 mm), while Laser 4 is close the ceiling (1550 mm from the floor, z = 625 mm).

**Figure 3:**
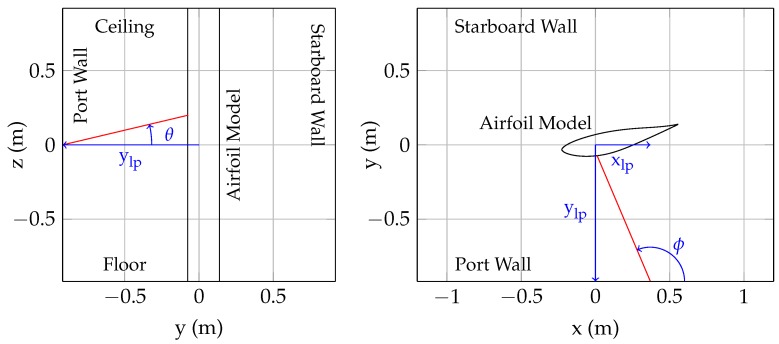
Definition of laser position/orientation, as seen looking upstream from inside the test section (**left**); and from above the test section (**right**).

These laser distance sensors were first used in the VT SWT in late 2014 [12]. Initially, a single laser was installed in the port wall at the Laser 2 location. The principle behind the initial laser measurement is shown in Figure 3. If the laser orientation (ϕ, θ) and position ($x_{\mathrm{lp}}$, $y_{\mathrm{lp}}$) relative to the fixed model rotation point are known, the model angle of attack can be inferred from the laser distance measurement and the model profile shape. These initial measurements showed that the encoder angle of attack (the angle set by the turntable) and the laser angle of attack (the angle inferred from the laser) were inconsistent, indicating that the model was deflecting or rotating. Three additional sensors (Lasers 1, 3, and 4) were then installed to further study model deflections and rotations.

## 3. Results

The following subsections describe the measurements required to implement the laser sensors in the VT SWT, from temperature compensation to position/orientation calibration. The end of the section details measurements the rotation and displacement measurements made with the DU96-W-180 model.

### 3.1. Sensitivity to Temperature

During the initial use of these lasers in the Stability Wind Tunnel [12], the wind tunnel operators noted that the distance readings from the laser would change slightly with temperature. The wind tunnel is subject to atmospheric conditions, so the air temperature in the wind tunnel is correlated to the outside air temperature. Additionally, as the wind tunnel is operated for long stretches of time, the air temperature will rise due to convective heating from the wind tunnel motor. Because small changes in distance correspond to significant changes in model angle of attack, the distance output from the lasers needed to be verified for varying temperatures, independent of model deflections and aerodynamic loading.

**Figure 4:**
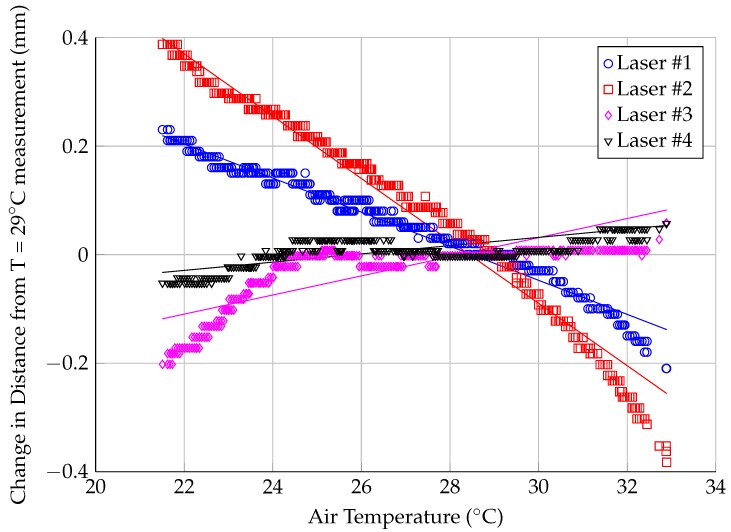
Sensitivity of laser distance sensors to changes in air temperature.

The results from an in-situ temperature calibration are shown in Figure 4. Static targets were placed in front of each laser inside of the test section. The laser distances and temperature were recorded every 25 s overnight, while the air temperature dropped from $33\;{}^{\circ }$C to $21{}^{\circ }$C. As the temperature dropped through the night, the distance readings from the laser changed. The exact cause of this temperature drift is unknown, but may be caused by thermal expansion of lenses and other mechanical components in the laser sensors. The temperature drift is large enough to cause a significant change in angle of attack. (A 0.8 mm change in Laser 2 would cause a ∼$0.13^{\circ }$ shift in the calculated angle of attack.)

The temperature drift was accounted for by applying a linear regression to the results shown in Figure 4. The temperature shift data were offset such that the average shift at $\left(29\pm 1\right)\;{}^{\circ }$C was zero; $29\;{}^{\circ }$C was chosen as the calibration temperature because the laser position calibration (described in the following subsection) was performed at this temperature. The parameters for the regression fits are shown in Table 1, while the regression is shown in Figure 4. The regression lines do not track the temperature drift across the entire temperature range, so there is added uncertainty in the measurement after considering the temperature drift. The maximum difference between the data points shown in Figure 1 and the regression line is shown in Table 1; this value is added to the uncertainty based on the resolution of the measurement when considering the total uncertainty in the measured distance for the remaining calculations in this paper.

### 3.2. Laser Position Calibration

Before the lasers can be used in a wind tunnel experiment, the position (and orientation) of the laser sensors needs to be determined. Once the orientation and position of the lasers is known, the laser readings can be used to track the location of the model within the test section. With the wind tunnel turned off (no air flow), the model was rotated between $-16^{\circ }$ and $16^{\circ }$ angle of attack in $1^{\circ }$ increments. At each angle, the angle of attack (as measured by the encoder) and the laser distances are recorded. A simplex optimization routine was then used to calculate the laser positions/orientations ($x_{\mathrm{lp}}$, $y_{\mathrm{lp}}$, θ, and ϕ, as shown in Figure 3) that reproduce the encoder angle of attack for all of the measurements, assuming that the model’s rotation point is fixed in space.

**Figure 5:**
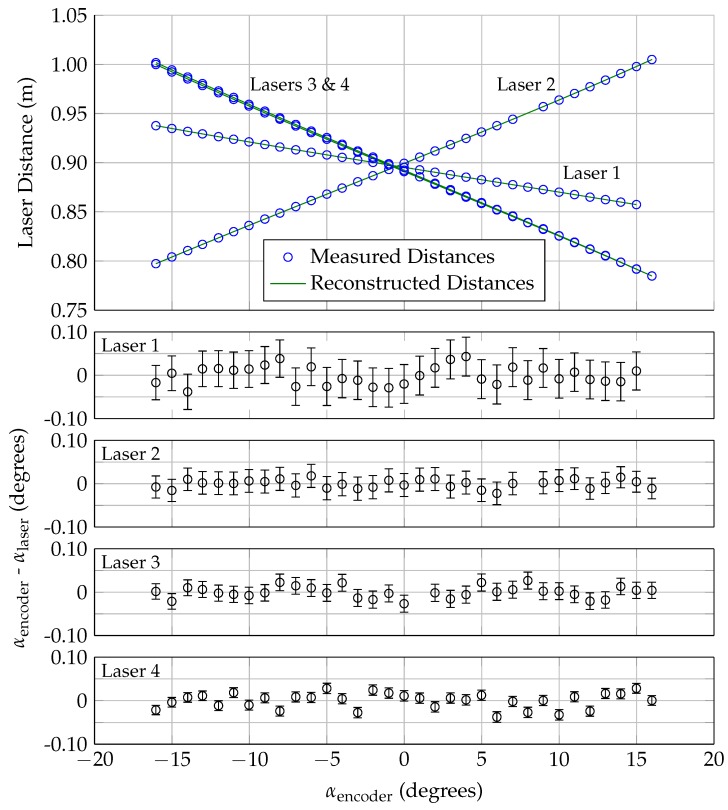
Laser position/orientation calibration data.

Results from this calibration are shown in Figure 5. The calculated laser positions/orientations (shown in Table 2) are consistent with the positions/orientations of the lasers in the wind tunnel. The top plot in Figure 5 shows the measured laser distance for all four lasers as a function of angle of attack, while the remaining four plots show the calibration residuals (the difference between encoder angle of attack and the reconstructed laser angle of attack, using the recorded distance and the calibration constants.)

The error bars represent the maximum uncertainty in the angle of attack. This uncertainty was calculated by first considering the uncertainty in the measured laser distance, which was calculated by combining the uncertainty due to the measurement resolution and the uncertainty due to the temperature correction, shown in the last column of Table 1. The maximum uncertainty in the angle of attack was then calculated by adding (or subtracting) the cumulative uncertainty in the distance to the measured distance reading, and calculating the associated change in angle of attack. Both the positive and negative uncertainty were calculated individually for each measurement point.

For the model geometry and laser locations used in this work, the relationship between laser distance and angle of attack was nearly linear for all four lasers in this angle of attack range. Laser 1 is the least sensitive to changes in angle of attack, because this laser is closer to the model rotation point than the other three lasers. The angle of attack uncertainty is nearly symmetric about the measured value and only varies weakly with the measured distance, because the relationship between laser distance and angle of attack is nearly linear. The largest uncertainties in the angle of attack results are ±0.042, ±0.027, ±0.020, and ±0.011 degrees for Lasers 1–4, respectively. Over 80% of the residuals are zero within the uncertainty of the angle of attack measurement, with the largest residuals being 0.044, 0.027, 0.020, and 0.011 for Lasers 1–4, respectively.

### 3.3. Rotation and Deflection Analysis

**Figure 6:**
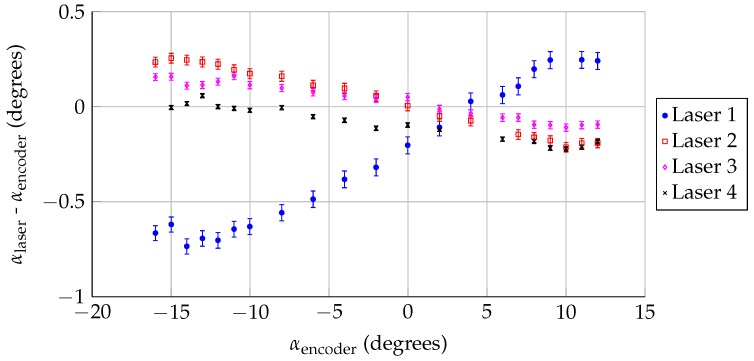
Difference between angle of attack measured by the lasers and the encoder, assuming that the model rotation point is fixed, for Rec = 3.0 million.

Once the laser positions/orientations were calculated, the sensors were used to track model angle of attack during a wind-on measurement, as described in Section 2.2. For this analysis, the center of rotation was assumed to not move during the test, but the model could rotate/twist under aerodynamic load. The difference between the encoder angle of attack and the calculated angle of attack from the lasers is shown in Figure 6. Lasers 2–4 (which are downstream of the model rotation point) show a difference in angle of attack that becomes more negative as the lift is increased. In contrast, Laser 1 (which is closer to and upstream of the model rotation point) shows the opposite trend. The discrepancy between Laser 1 and Lasers 2–4 is an order of magnitude larger than the uncertainty in the measurement, and indicates that the model is not uniformly rotating, but deforming in a more complex way. The model could be shifting or bending, which would move the model rotation point as a function along the span.

**Figure 7:**
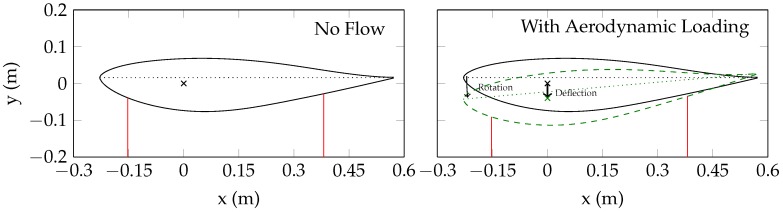
Displacement/rotation analysis using Lasers 1 and 2.

Lasers 1 and 2 were placed at the same spanwise location to address the discrepancy shown in Figure 6 and separate deflection/bending from rotation. Figure 7 shows the concept behind this calculation. In this analysis, the model rotation is allowed to move in the wall-normal direction. The distance output from the two lasers (two inputs) is used to solve a system of non-linear equations for two outputs: the rotation and the translation. By convention, a positive translation is movement towards the starboard wall, and a positive rotation is an increase in the angle of attack.

This analysis provides the rotation and deflection at a single spanwise location. Lasers 3 and 4 can then be used to gain information about the model’s position at different spanwise locations. Because there is only one laser at these spanwise locations, the displacement and rotation cannot be determined independently. For this analysis, we assumed that the airfoil model is rotating but not twisting, or that the angle of attack does not vary across the span. (This assumption is based on the shape and construction of the model.) If this assumption is true, than the distances from Lasers 3 and 4 can be used to determine the model deflection at each spanwise location.

**Figure 8:**
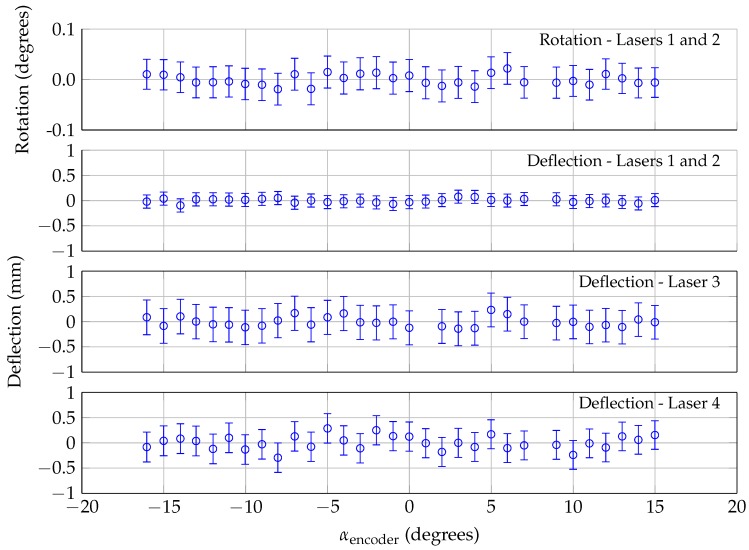
Analysis of laser calibration data (with wind off), using displacement/rotation analysis.

This deflection/rotation analysis was applied to the calibration data shown in Figure 5 to quantify the uncertainty associated with this technique. Because there was no aerodynamic loading during the calibration, the rotation and deflection should be identically zero. The results from this analysis are shown in Figure 8. The error bars represent the maximum uncertainty in the rotation/deflection, which is calculated independently for each measurement point. For the rotation/deflection, the uncertainty was calculated by finding the worst-case scenario from different combinations of adding/subtracting the cumulative distance uncertainty to the measured distances from Lasers 1 and 2. The deflection uncertainty for Lasers 3 and 4 was calculated in a similar manner, while also incorporating the uncertainty in the rotation calculation. As noted in Section 3.2, the error bars are nearly symmetrical and only vary weakly with angle of attack, because the relationships between laser distance and angle of attack for zero deflection are nearly linear (Figure 5).

As expected, the rotation and deflections were zero within the uncertainty of the calculation. The uncertainty in the rotation was ±0.032 degrees, and the uncertainty in the deflection calculated by Lasers 1 and 2 was ±0.13 mm. The uncertainty associated with the deflection measured by Lasers 3 and 4 was ±0.34 mm; the uncertainty for these calculations is larger because of the added uncertainty in calculating the model rotation (which is then used to calculate the deflections measured by Lasers 3 and 4.)

### 3.4. Model Deflections/Rotations under Aerodynamic Loading

The rotation/deflection analysis described in Section 3.3 was applied to 13 sets of DU96-W-180 aerodynamic measurements (8 clean and 5 tripped) in the VT SWT, taken over a two-week period. All of these runs were performed at a chord Reynolds number of 3.0 million. The air temperature varied from run to run, between $23.5\;{}^{\circ }$C and $33.4\;{}^{\circ }$C; the flow speed was adjusted to maintain a Reynolds number of 3.0 million, leading to flow speeds between 62.7 m/s and 66.7 m/s.

**Figure 9:**
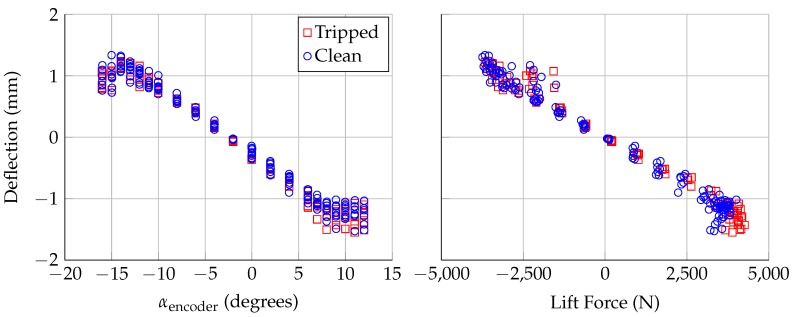
Deflection measured using Lasers 1 and 2, plotted against: encoder angle of attack (**left**); and applied aerodynamic lift force (**right**). Deflections are relative to deflection at $\alpha_{l=0}$. Uncertainty in deflection is ±0.13 mm.

Figure 9 shows the deflection calculated using the distances calculated from Lasers 1 and 2. Figure 2 shows that there is zero lift force on the model near $-2.5^{\circ }$ angle of attack; however, Lasers 1 and 2 showed a wide range of deflections at this angle, from −0.65 mm to 0.73 mm. This suggests that there might be some small shifts in model position over time. For this plot, the zero lift deflection was subtracted out to show the deflection relative to the zero lift deflection. At this spanwise location (660 mm above the floor), the model deflects up to 1.5 mm in the direction of the applied lift. The deflection is a linear function of the load in the linear region of the lift curve, from $\alpha =-10^{\circ }$ to $\alpha =8^{\circ }$. This deflection explains why Figure 6 shows discrepancies between the laser outputs; the model rotation point is not fixed in space, and multiple lasers need to be used to calculate the model angle of attack.

**Figure 10:**
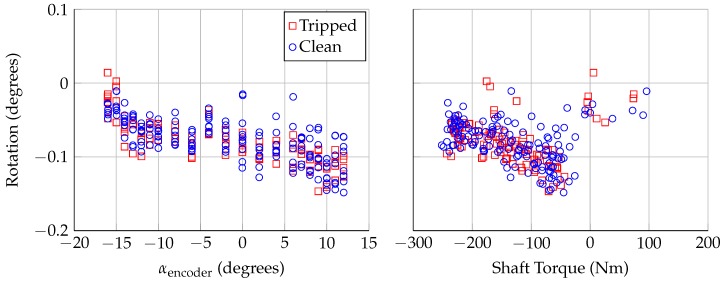
Rotation calculated using Lasers 1 and 2, plotted against: encoder angle of attack (**left**); and aerodynamic torque applied to model mounting shaft (**right**). Uncertainty in rotation is $\pm 0.032^{\circ }$.

Figure 10 shows the rotation calculated by Lasers 1 and 2, as a function of the the encoder angle of attack and the applied torque on the model shaft. The model rotation is approximately 0${}^{\circ }$ at the most negative angle of attack, when the shaft torque is near-zero or slightly positive. As the angle of attack is increased, to $-14^{\circ }$, there is a large shift in shaft torque to approximately −250 Nm, and the model undergoes a slight negative rotation. As the angle of attack is increased further, the magnitude of the negative rotation increases, to approximately $-0.11^{\circ }$ at the largest angles of attack. The relationship between the shaft torque and rotation shown in Figure 10 was not anticipated; the expectation was that more negative shaft torques would create larger negative rotations. The torque/rotation points show the opposite trend, but it should be noted that the uncertainty in the rotation is $\pm 0.032^{\circ }$.

**Figure 11:**
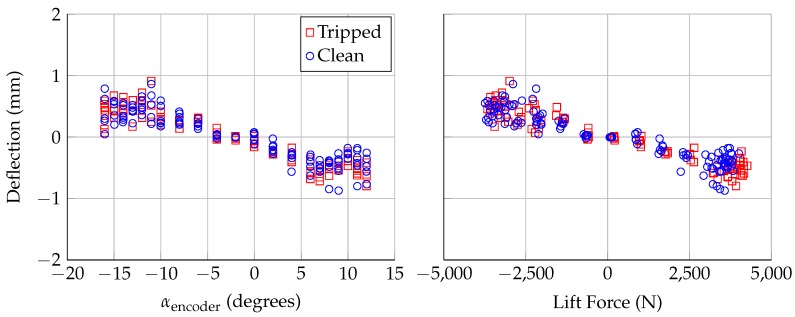
Deflection at span position of Laser 3, assuming no twist, plotted: against encoder angle of attack (**left**); and aerodynamic lift force (**right**). Uncertainty in deflection is ±0.34 mm.

**Figure 12:**
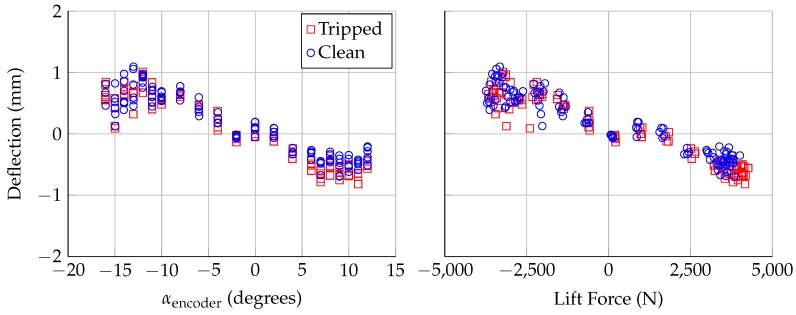
Deflection at span position of Laser 4, assuming no twist, plotted against: encoder angle of attack (**left**); and aerodynamic lift force (**right**). Uncertainty in deflection is ±0.34 mm.

The deflections calculated from Lasers 3 and 4 provide evidence that the model is bending under load. Figure 11 and Figure 12 show the calculated deflections from Lasers 3 and 4, respectively. As with Figure 9, the deflection at zero lift was subtracted out to show the relative deflection at these spanwise locations. Both Lasers 3 and 4 show deflections of ±0.8 mm, which is larger than the uncertainty in the calculated deflection (±0.34 mm). The model is deflecting in the direction of the applied lift, which is consistent with the deflection calculated using Lasers 1 and 2. Under maximum loading, the model is deflecting ∼1.5 mm near mid-span and ∼0.8 mm close to the floor and ceiling; this deflection is consistent with model bending, with the model fixed at the floor and ceiling.

**Figure 13:**
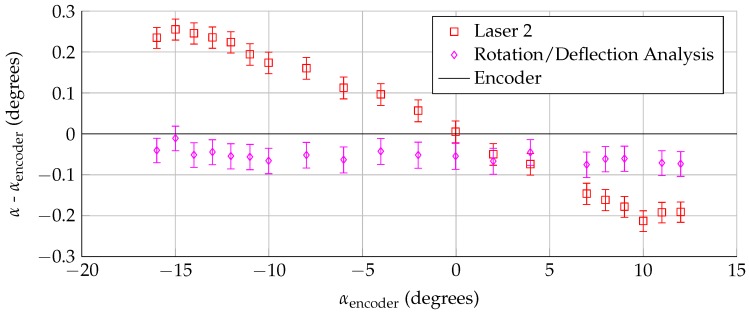
Comparison of angle of attack measurements for a single run at Rec = 3.0 million.

Finally, Figure 13 shows the rotation/deflection analysis for the same dataset shown in Figure 6. The figure shows the difference between the angle of attack measured by the encoder and two methods discussed in this paper: using a single laser while assuming that the center of rotation is fixed, and using multiple lasers at the same span location to account for model bending/translation. The multiple-laser technique shows that the model rotates slightly under loading, creating an approximately $-0.07^{\circ }$ offset in the angle of attack. This technique also showed a small amount of model bending (<2 mm). Previous studies with this model [12,13] have shown that boundary layer transition and other aerodynamic qualities do not vary in the span (z) direction, which suggests that this model bending does not affect the model aerodynamics. However, the bending is enough to bias the single laser angle of attack measurement. For example: at $-14^{\circ }$ encoder angle of attack, using only Laser 2 to calculate the angle of attack (while assuming that the center of rotation point is fixed) leads to an angle of attack measurement of $-13.75^{\circ }$. When Lasers 1 and 2 are used simultaneously to remove bending effects, the angle of attack measurement is $-14.05^{\circ }$. This shows that the bias caused by model bending can be even larger than the measured rotation, and multiple lasers should be used to accurately measure the angle of attack.

## 4. Summary and Conclusions

In this work, the authors installed laser displacement sensors in the walls of a wind tunnel to measure the position of airfoil models under aerodynamic loading. Four commercially available Acuity AR700-32 triangulation sensors were integrated into the wind tunnel data acquisition system; these lasers tracked the position of the model in the test section as the model rotated/deflected under load. The distance output from the lasers was found to drift slightly with temperature; this drift was accounted for using a linear regression from an in-situ temperature calibration. Two lasers near mid-span showed that the model was undergoing a small rotation (<0.15${}^{\circ }$) under aerodynamic load. The combination of all four lasers was used to confirm that the model is bending slightly under load, with maximum bending deflections under 2 mm. Special care was taken to calculate the uncertainties in the measured rotations and deflections, based on the resolution of the laser sensors and the uncertainty in the applied temperature compensation.

Laser distance sensors were chosen over other measurement techniques (such as accelerometers or photogrammetry) because they offer the potential for an absolute position reference. Ideally, this means that the lasers could remain installed in the wind tunnel and be used to establish the $0^{\circ }$ position of any model installed in the wind tunnel. In practice, this is complicated by drift in the distance output from the laser sensors, both as a function of temperature (as shown in Figure 4) and as a function of time. Because a typical wind tunnel test lasts multiple weeks, further tests need to be performed on the laser sensors to quantify the stability of the output over long time scales, both independently of temperature and in-situ with temperature changes. Some of this drift can be calibrated out using the procedure described in Section 3.2, but quantifying the drift will provide a better understanding of the limitations of these sensors for this application.

## Figures and Tables

**Table 1:** Temperature compensations fit parameters and uncertainty.

Laser	Slope (mm/${}^{\circ }$C)	Offset at 29 ${}^{\circ }$C (mm)	Maximum Difference (mm)
1	−0.031	−0.016	0.072
2	−0.057	−0.032	0.127
3	0.018	0.014	0.088
4	0.008	0.024	0.038

**Table 2:** Laser position/orientation values from calibration.

Laser	$x_{\mathrm{lp}}$, (m)	$y_{\mathrm{lp}}$, (m)	θ (degrees)	ϕ (degrees)
1	−0.1743	−0.9307	0.01	89.14
2	0.3712	−0.9301	−0.01	90.20
3	0.3731	0.9273	0.00	−89.59
4	0.3828	0.9280	0.05	−89.94

## Abbreviations

The following abbreviations are used in this manuscript:

VT SWT	Virginia Tech Stability Wind Tunnel
